# The Girl Part of the Boy Problem

**Published:** 1913-10

**Authors:** Eugene C. Foster

**Affiliations:** City Secretary for the Boys in the Detroit Y. M. C. A.


### The Girl Part of the Boy Problem.

BY EUGENE C. FOSTER, CITY SECRETARY FOR THE BOYS IN THE DETROIT Y. M. C. A.

Do girls and women realize what a part their dress plays in the life of the world about them? There would be a swift and radical change in the dress of some, if they could see the influence upon boys and men of their present mode of dressing. Probably not before in this generation have there been any such prevailingly offensive styles in the women's dress of America as there are today. Need it continue? Mr. Foster's frank words are so sorely demanded! may there be a genuine response to this protest for the safeguarding of lives into which evil now finds so ready an access.

The growing boy has been under the searchlight of investigation in a remarkable way, especially within the past ten years. So far as he is concerned we may frankly say that he is no longer the formidable "problem" that he was once supposed to be.

One of the most significant things that has been brought home to us with emphasis is the fact that the problems of boyhood are intricately involved with other relationships, and not the least of these is the relationship of the boy and the girl. It is my privilege to do my thinking in terms of thirty thousand boys in the teen age, the entire boyhood of a great city. Not that I have relationship with any such number; but along with definitely constructive duties I am set as a watchman upon the wall to sound the alarm when any danger seems to menace the boyhood life of the community. This danger makes me of necessity a student of boyhood life in large cities, and I have a mind to write with earnest frankness concerning a phase of this girl element that appears to be a new menace—impossible as it has been proved to be for anything to be really new.

I refer to the prevailing manner of dress among women, more especially as seen upon our city streets. I have no hesitation in saying, that it is not alone immodest, but it is as well immoral; and I greatly fear that we shall soon see an overwhelming torrent of moral laxity engulf our youth. Indeed, it is here.

I have never known a time in our cities when the young man intent upon picking up acquaintance with a girl on the street might not be reasonably sure of accomplishing this with a small amount of effort in certain fairly well-defined sections; but never in all by observation, until lately, have I known a time, when, by day or night, in thoroughfares devoted to shopping or business, a procession of girls sweep past a young man in dress so vulgar that he might fairly—even though mistaken—assume that they are on parade to invite his advances. The dress of the girl of today causes her to be thrust upon him, perhaps at times when his own thoughts are far from the realm of ungentlemanly conduct, and perhaps with no desire on her part to arouse such interest.

The seventeen-year old boy who sat in my office a few nights ago and recounted the struggles of a young man to keep himself pure, was, unfortunately, but a type of many; and the girl of chance street acquaintance who caused him to lose his fight is but a type too. That is sad enough; but one bows his head in shame when the boy says, "How did I know she wasn't decent? Hundreds of girls on the streets dress and act like she did."

A man of considerable experience recently designated a certain country hotel as a "low resort." He was promptly challenged by another, who happened to be a circuit court judge. The first man defended his criticism by citing the women who were seen at this hotel; whereupon the judge remarked: "You are wrong. The women who go there, go with their husbands, and are respectable matrons of the community. You fail to make allowance for the present immodesty in women's dress; an immodesty which causes me to be ashamed to meet some of my most respected woman acquaintances on the streets of our city."

Unhappily, the judge was right. And right here is the crux of the difficulty. If only immodest women and girls dressed in vulgar fashion, the line would be sharply drawn. But this immodest dress prevails among all classes. Those of us who give our lives to boys feel no harder task than to help the boy in his battle to keep pure. Imagine such a boy fighting such a terrific battle as only a man can appreciate, confronted not only once but a hundred times with indecencies in dress as he walks a few blocks in the heart of a city! Will he win or lose?

I do not write as a fashion critic; I write as a man who daily faces the moral issues raised by these things. But I want to be specific. There are prevailing styles of dress which are offensively immodest. Among these are: the tight-fitting waist; the "peek-a-boo" waist, in its really offensive forms (a common object of jest, while it goes on sowing seed for its unhallowed harvest); some styles of low neck and short sleeves which many girls affect; many forms of tight fitting skirts; offensively short; certain types of hosiery. The list might be made longer; it is merely used by illustration.

Women are crying out because of the liberties among our men. I believe their cries rise to heaven, and that heaven weeps over the shame of it. But I say without hesitation that those prevailing styles of dress are loosing the passions of countless thousands of growing boys whose physical fight is already severe.

When will women understand? I make no charge of indecent design against thousands of women whose thoughts and lives are far above such a thing. Granted that they are above such thoughts; will they not listen to those who know these things?

Two girls of irreproachable character passed me on the street but a day or two since; they were dressed conspicuously and, I should say, immodestly. The crossing policeman caught the eye of a teamster and winked, and the teamster replied with a sneering smile. The girls never knew of the estimate those two men placed upon them.

Just ahead of me the other day walked a young woman whose face, apart from her costume, betokened refinement; but her dress was of the extreme close-fitting type, with low neck and short sleeves. Young men behind me spoke in the coarsest terms of her; others stopped and stared; still others turned about and walked away in her direction to get a better look. Apparently she was unconscious of the fact that with her appearance on the street the ideals of womanhood were lowered in the minds of any men.

But it does not stop there. These women of better circumstances set the pace absolutely for the girl of small wages. The girls may have no home to which her friends may be invited; her social effort is expended in dress. She follows the prevailing fashion of immodesty; she inflames the passion of the young men she meets; she may not be sheltered and safeguarded; and she is swept under.

Is there a cure for it all? It is a woman's problem. If going to the extreme in dress is more important in the eyes of womankind than safeguarding the pathway of sons, brothers, husbands, then it will continue. But those who see it as it is must raise their voice in protest.—*Maryland White Ribbon Herald.*

